# BCG skin reaction in Mantoux-negative healthy children

**DOI:** 10.1186/1471-2334-5-19

**Published:** 2005-03-29

**Authors:** Mohit Singla, Vaibhav Sahai, Sukhbir Sodhi, Rakesh Pal Gupta

**Affiliations:** 1 Section of Immunobiology, Yale University School of Medicine, 300 Cedar Street, TAC S 630, New Haven, CT 06510, USA; 2 Medical Internist, Government Medical College, Amritsar, India; 3 Department of Pharmacology, Himachal Dental College, Sundernagar, India; 4 Department of Pediatrics, Singla Hospital, Hoshiarpur, India

## Abstract

**Background:**

Tuberculosis poses a great challenge, especially in children. The response of BCG Test may be different in previously vaccinated children and needs to be considered before interpreting positivity for TB. This study has been carried out to determine the pattern of BCG reaction comparing previously vaccinated with non-vaccinated children.

**Methods:**

The study was conducted in the healthy school children aged 4–6 years. The BCG skin reaction in Mantoux-negative children was compared between children with and without previous BCG scar. After the Mantoux and BCG Test, the analysis of variance was done as per protocol.

**Results:**

Out of 50 children previously BCG vaccinated, 39(78%) showed exaggerated BCG test responses while out of another 50 children who were not vaccinated for TB, only 9(18%) showed exaggerated BCG Test response (p-value < 0.00001). Average induration obtained in children who were immunized with BCG at birth was much greater than those who were not immunized. 80% and 76% males and females respectively in Group I showed exaggerated BCG response while 16% and 20% males and females respectively of Group II showed exaggerated BCG response.

**Conclusion:**

The present study indicates that normal healthy children may have a mild exaggerated BCG Test response i.e. induration up to 8 mm because of prior BCG vaccination. Therefore, BCG Test, though important should not be the only criteria for start of chemotherapy for TB in children as the side effects of drugs may cause much morbidity. An induration up to 8 mm after the BCG Test can be normal in Indian settings due to exposure to Mycobacterium in environment and/or BCG vaccine.

## Background

Tuberculosis remains a leading cause of death from any single infectious disease, accounting for over a quarter of all avoidable deaths. It infects about 1 billion people and causing an estimated 1–2 million deaths annually [1]. Nearly 3–4 million children in India have tuberculosis while 94 million are at risk to infection. 40% of these children by the age of 6 yrs and 80% by the age of 16 years are labeled as infected [2].

Bacillus Calmette-Guerin (BCG) vaccine, which consists of live attenuated Mycobacterium tubercle bovine Danish 1331 strain, has been extensively used as a protective measure against tuberculosis for the last half century.

Under Universal immunization Programme (UIP), primary BCG vaccination is given at birth or within the first month. The attenuated bovine non-pathogenic Mycobacterium induces tuberculin sensitivity and potentates the defense mechanism enabling the recipient to combat re-infection when exposed to pathological strains of mycobacterium later. This primary infection enables the vaccinated person to mobilize immune processes more rapidly when challenged by further natural infections.

A definite scar evidences an effective BCG vaccination and absence of this denotes that no immunity is attributable to the vaccination [14]. However, even with proper vaccination the immunity has been observed to decline within a few years, on the basis on which revaccination at five years has been suggested as an optional dose by the Indian Academy of Pediatrics [6]. Vijayalakshmi et al[11] suggested that revaccination may be contemplated after 8 years.

BCG given as a diagnostic test is based on Koch's phenomenon. When BCG is given to a child with tuberculosis, the reaction occurs at the site of vaccination within 48–72 hrs as against the usual late reaction i.e. after 3–6 weeks in child without tuberculosis. The reaction is measured with a standard plastic scale and the maximum size of the reaction is noted. The appearance of papule or induration more than 5 mm in size at the test site is considered as positive BCG Test [7-11].

BCG Test response is graded as mild (5–10 mm), moderate (10–20 mm) or severe (more than 20 mm) [6]. The reaction is complete if all the stages i.e. papule, pustule, ulcer and scab are seen, but is incomplete when only a papule or nodule is seen and the reaction does not progress to a stage of pustule or scab formation.

BCG response is more sensitive than the Mantoux test (Tuberculin test) and hence has been considered a better tool for epidemiological investigations. However, the effect of previous BCG vaccine on the subsequent revaccination is not well established and only a few elaborate studies have been carried out in this regard. It has been seen that the Mantoux test is not reliable as a post-vaccination check, because in vitro, cell-mediated immune responses may be demonstrable even when Mantoux test becomes negative [11,12].

The response of BCG may be different in previously vaccinated children and needs to be considered before interpreting positivity. The exact type of reaction and range of induration is not clear cut or well established.

There is possibility of over diagnosis of tuberculosis on the basis of BCG Test results, if previous BCG vaccination status is not taken into consideration. It is to be noted that the BCG Test may be positive in previously vaccinated children even when Mantoux test is negative.

This study has been carried out to determine the pattern of BCG reaction comparing previously vaccinated with non-vaccinated children.

### Aims and objectives

1. To assess the BCG skin reaction in Mantoux-negative healthy children at 4–6 years of age previously vaccinated with BCG, as evidenced by the presence of scar [14]

2. To compare the reaction between children with and without previous BCG scar.

## Methods

The study was conducted to determine the BCG response in 100 healthy, Mantoux-negative children in the age group of 4–6 years in relation to previous vaccination status. The informed consent of the parents was taken. Basic information, including the immunization status, was obtained on a written proforma from the parents of 193 children of 4–6 yrs age group belonging to kindergarten schools of urban and rural areas of Ludhiana. The immunization status of the children was confirmed by the presence of BCG scar[14]. Children with measles or other serious infections in previous 6 months were excluded. Also, only those with body weight above 80%of reference weight (50th percentile of the Harvard standard for the chronological age) were selected. Children presenting with clinical features of any disease, including tuberculosis, were excluded.

Mantoux test was given by injecting 0.1 ml (5 TU) of PPD intradermally on volar surface of left forearm. The test was read after 48–72 hrs and induration of 5 mm or less was taken as negative. Those with induration above 5 mm were excluded. The children were divided into two groups on the basis of previous BCG vaccination.

Group I: BCG given at birth.

Group II: BCG not given at birth.

The first 25 boys and 25 girls in Group I and Group II each who fulfilled the above criteria were included in the study. The children in both groups were given the BCG Test and the reaction was observed for erythema and induration after 1 day, 3 days, 7 days and 6 weeks as detailed below. Children in the Group I with induration 10 mm or more and in Group II with 6 mm or more were further investigated with x-ray chest to rule out any chance of tuberculosis and thus eliminate false positives in the study.

### BCG test

Freshly diluted freeze-dried 3CC vaccine were mixed. Dry vaccine and solvent was carried from the refrigerator in an icebox to the place of vaccination. 0.1 ml of the solution containing 0.1 mg BCG vaccine was administered intradermally in the deltoid region using sterile disposable needles.

### Observation of Reaction

The reaction was observed after 24 hrs, 72 hrs, one week and 6 weeks. The horizontal and vertical diameters of induration were measured. All the measurements were made by non-stretchable white fiberglass tape measuring to the nearest of 1 mm. The reactions in the two groups were recorded.

## Results

Out of 50 children in Group I (children who were given prior BCG vaccination), 39 (i.e. 78 per cent) had exaggerated BCG Test responses while in Group II (children who were not given prior BCG vaccine) only 9 (18 per cent) out of 50 cases had exaggerated BCG Test response (Refer to Table 1). Critical difference at 5% of 0.64 and F-ratio of 60.4 indicates high statistically significant difference between the two groups.

**Table 1:** Comparison of Exaggerated BCG response in two groups

	Number of cases			Exaggerated response cases					
	
Study Group	Male	Female	Total	Male		Female		Total	
				
				Total	%	Total	%	Total	%
Group I	25	25	50	20	80	19	75	39	78
Group II	25	25	50	4	16	5	20	9	18
Total	50	50	100	24	48	24	48	48	48

When the exaggerated BCG Test response cases in Group I were further evaluated for size of the induration, it was seen that 72% cases had induration between 6 to 8 mm (Refer to Table 2). Only 2 cases had induration of greater or equal to 10 mm. These two cases were further investigated with x-ray chest to rule out the possibility of tuberculosis. Similarly in Group II, 12% had exaggerated BCG test response of 6 mm whereas rest 6% had 7 mm induration.

**Table 2:** Exaggerated BCG response in relation to scar size

		Exaggerated response	
		
Scar size	Total cases	Total	%
No scar	50	9	18
Faint scar	27	21	77
Good scar	11	8	72
Large scar	12	10	83

Almost same number of males and females in both groups had exaggerated BCG response (Refer to Table 1). No statistically significant difference was observed between males and females in either of the two groups.

Highly significant statistical difference of exaggerated BCG response between Group I (BCG given at birth) and Group II (BCG not given at birth) were obtained.

To test the significance of this difference of the exaggerated response to BCG Test between Group I and Group II, a Standardized Normal Test for Proportions (Z-Test) was used. For the calculated value of Z = 7.518, the p-value is less than 0.00001; thus the exaggerated response of Group I was found to be highly significant in comparison to that of Group II.

## Discussion

BCG has been extensively used as a protective measure against tuberculosis for the last half a century [5]. In the previous two decades encouraging results have been reported regarding BCG vaccination as a diagnostic test for tuberculosis and it has been termed BCG Test [3,4].

The effect of previous BCG on subsequent revaccination response is not well established and only a few elaborate studies have been carried out in this regard. It has been seen that Mantoux test is not reliable as a post-vaccination check; because in-vitro cell mediated immune response may be demonstrable even when Mantoux test becomes negative [11,12].

The response of BCG Test may be different in previously vaccinated children. It needs to be reconsidered before interpreting postivity. The present study was carried out to determine the pattern of BCG reaction comparing previously vaccinated with non-vaccinated children.

As already mentioned very few studies regarding BCG Test in prior BCG vaccinated children have been carried out. One such study is by P.M.Udani[7]: BCG Test in the diagnosis of tuberculosis in children – Indian Pediatric, (1982) addendum regarding "BCG Test in children who have received prior BCG is available." In this study he arrived at three sets of conclusions.

### First

Negative BCG Test: the infant or child may not get any reaction because of BCG vaccination and body behaves as if no prior immunization was given. The prior BCG vaccine given at birth did not contribute to immune response of the child.

### Second

Strongly positive BCG Test, a classical accelerated reaction. This reaction is similar to reaction seen in a patient having tuberculous disease.

### Third

Mildly positive reaction. The child may get induration between 6–9 mm in this reaction. He attributed the third type of reaction because of prior BCG vaccination.

In the present study, most (about 84%) of the exaggerated BCG Test response in Group I had reaction between 6–8 mm similar to the Third type of reaction evidenced by Udani[7]. In the study conducted by Udani[7], no inclusion and exclusion criteria for study have been mentioned, and probably all children who were given prior BCG vaccination were included in the study. As mentioned earlier the present study compares the results of the BCG Test in BCG vaccinated children with BCG non-vaccinated children.

Although the 50 boys and 50 girls who were included in this study were not exactly selected on the basis of formal random sampling procedures, there is little possibility of bias. Even though there was no entry criterion, except for the body weight above 80% and exclusion of any case with any disease process, we understand that the absence of formal random allocation is a limitation to the study. However, the highly significant results (p-value < 0.00001) considerably overshadow the limitation.

Diagnosis of TB in children is not easy. Like in adults one should not always think of Therapeutic Trial in children as the drugs are very toxic and the side effects like Optic Neuritis cannot be easily diagnosed in children. So Diagnosing and treating TB in Children is a double-edged sword. These days PCR and other new investigations have become useful, but they just add to the old regime of investigation and are not fully predictive of disease on their own. So coming to a diagnosis of TB in children need a lot of clinical skills and interpreting the investigations in the right way.

There is no doubt that in some children it may be difficult to decide whether BCG Test is positive because of prior BCG vaccination or due to natural tubercular infection. Over diagnosis of tuberculosis is usually made by using exaggerated BCG Test response as a parameter to decide anti-tubercular therapy in patients in whom otherwise history, clinical features, tuberculin test sensitivity, x-ray chest, etc. are not fully suggestive of the Koch's disease. The present study indicates that normal healthy children may have a mild exaggerated BCG Test response i.e. induration up to 8 mm because of prior BCG vaccination. Therefore, the patients who were immunized with BCG at birth and tested positive for BCG Test, and in whom other parameters for diagnosis of tuberculosis are not suggestive, are perhaps receiving unnecessary anti-tubercular therapy. A positive test may be of value after correlating it with the age of the child, nutrition, size of tuberculin test induration, history of contact and symptoms to diagnose and institute chemotherapy [1].

## Conclusion

78% (39 out of 50) of children in Group I (prior BCG vaccination given) and 18% (9 out of 50) in Group II (prior BCG vaccination not given) had exaggerated BCG response; defined as induration greater than or equal to 6 mm. Statistical difference (p-value<0.00001) between Group I and Group II was highly significant. 72% of children in Group I, who showed exaggerated BCG response, had induration in range of 6–8 mm. Only 6% of children showed induration of 9, 10 or more than 10 mm. 22% of children in Group I had induration less than 6 mm. Children in Group II showing exaggerated BCG response had induration of either 6 or 7 mm. Differences between male and female readings in each of two groups were statistically insignificant. Day 1 and 3 readings were statistically more significant as compared to day 7 and 6-week readings. All children in Group II showing exaggerated BCG response and in Group I showing induration of 10 mm or more were investigated with x-ray chest to rule out tuberculosis. Even in absence of prior BCG vaccination, some normal healthy children of Group II exhibited mildly exaggerated BCG response. This can probably be explained on the basis of tubercular infection not warranting treatment. Significant number (78%) of normal children with previous BCG vaccination, who were Mantoux negative and received BCG as a 'diagnostic test' or as revaccination, showed, exaggerated BCG response. Since these children are healthy, an exaggerated BCG response in such a situation must not be construed as an evidence of tubercular disease.

## Abbreviations

BCG – Bacillus Calmette and Guerrin

## Competing interests

The author(s) declare that they have no competing interests.

## Authors' contributions

MS carried out designed the study, conducted all the fieldwork and drafted the manuscript. VS has participated in the statistical analysis and editing of the script. SSS helped in co-ordination and draft of the manuscript. RPG conceived the study and provided guidance in carrying out the work.

## Pre-publication history

The pre-publication history for this paper can be accessed here:


